# Meta-Analysis of Pollen Limitation Reveals the Relevance of Pollination Generalization in the Atlantic Forest of Brazil

**DOI:** 10.1371/journal.pone.0089498

**Published:** 2014-02-21

**Authors:** Marina Wolowski, Tia-Lynn Ashman, Leandro Freitas

**Affiliations:** 1 Programa de Pós-Graduação em Botânica, Jardim Botânico do Rio de Janeiro, Rio de Janeiro, RJ, Brazil; 2 Department of Biological Sciences, University of Pittsburgh, Pittsburgh, Pennsylvania, United States of America; 3 Jardim Botânico do Rio de Janeiro, Rio de Janeiro, RJ, Brazil; Lakehead University, Canada

## Abstract

Despite the extensive knowledge of pollen limitation in angiosperms, its assessment within tropical forests is still limited. Especially lacking are large scale comparisons of species within this biome – one that is highly diverse but also becoming increasingly threatened. In fact, many tropical plant species depend upon pollinators for reproduction but evaluation of the impact of this dependence via different levels of pollination specialization has yet to be made at the biome scale. We assessed the occurrence and magnitude of pollen limitation for species in the Brazilian Atlantic forest and tested the association of pollination specialization, breeding system, and life habit with pollination efficiency. We compiled data from studies published between 1985 and 2012. We calculated species' effect size (*d*) from data on fruit set after hand cross-pollination and natural pollination and conducted standard and phylogenetically independent meta-analysis. Overall pollen limitation was moderate, with magnitude of 0.50, and 95% confidence interval [0.37, 0.62] for 126 species. Pollen limitation was observed in 39% of species. Pollination specialization was the factor that best explained the occurrence of pollen limitation. Specifically, phenotypic and ecological specialists (plants with zygomorphic flowers and pollinated by one species of pollinator, respectively) had higher pollen limitation than generalist plants (actinomorphic flowers and pollination by two or more species). Functional generalists (plants pollinated by three or more functional groups) were not pollen limited. On the other hand, breeding system and life habit were not associated to pollen limitation. Pollen limitation was observed in the Atlantic forest and its magnitude was comparable to that for angiosperms as a whole. The finding that pollination specialization was the strongest predictor of pollen limitation suggests that specialist plants in this biome may be most prone to the reproductive failure as a result of pollinator loss.

## Introduction

Several synthetic and quantitative reviews have assessed the frequency and degree to which flowering plant reproductive performance (seed or fruit set) is reduced by inadequate receipt of pollen on the stigma, i.e., pollen limitation of reproduction (PL) [1]–[4]. Drawing on large data sets of angiosperms (e.g., 306 species [4]), these studies have established that PL is widespread. And while these syntheses have included a broad range of biomes (e.g., forests, grasslands, deserts), only a small percentage (15% of studies [3]) of the studies reviewed were conducted on species in the tropics. However, one review suggests that tropical species may be more prone to PL than temperate ones (e.g., the subset of self-incompatible species [2]). This may be due to the fact that more tropical species are animal-pollinated than temperate species (94% *versus* 78% [5]), or that plants in tropical forests tend to be outcrossing and have low density of adults (at least among trees [6]–[10]) – both phenomena that may place a premium on efficient cross-pollination to achieve maximal reproductive success. Moreover, the tropics support high biodiversity and pronounced levels of endemism [11], and their forests are severely threatened by human activities, for instance, by logging and agriculture [10]. In fact, PL has been seen to increase with plant diversity [12]–[13] and even more so for endemics [14]. The effects of diversity may result from greater interspecific competition for pollinators [4], [12] or greater heterospecific pollen transfer [14], while those of endemism may derive from smaller population sizes, reduced density and/or stronger habitat specificity [14]–[16]. The variation in PL for plants within tropical forests has not been widely assessed at least not at the biome scale. Such an analysis, however, will be informative as the putative causal traits can be assessed in the context of the shared evolutionary history of the tropical flora, and their ecological interactions with fauna.

**Figure 1:**
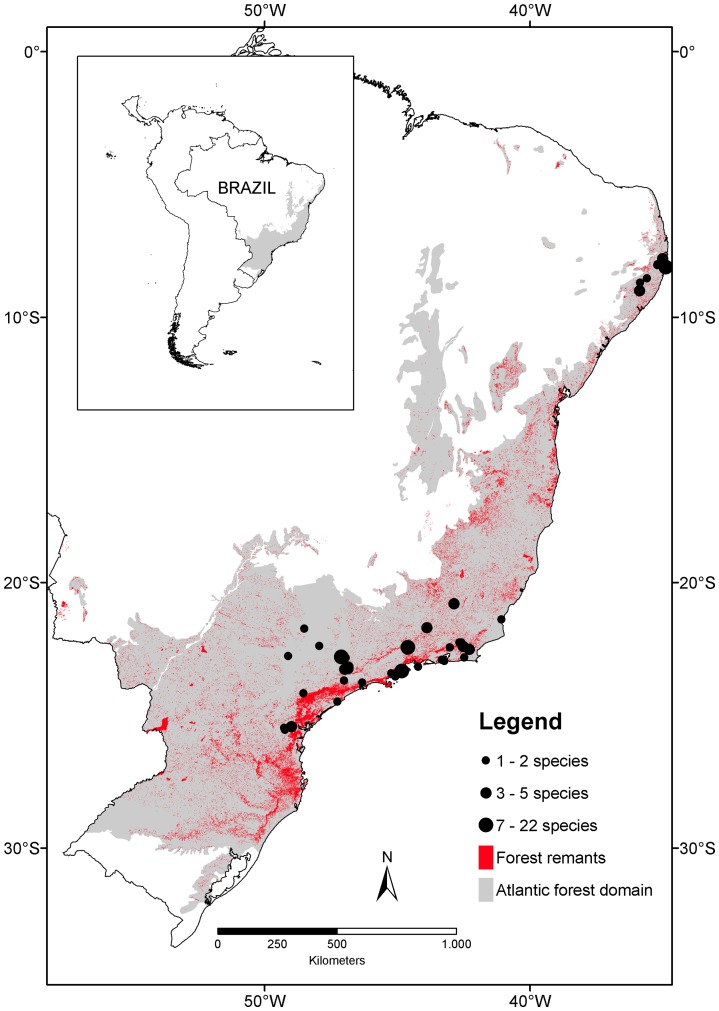
Geographic distribution of the species included in the meta-analysis. Map of Brazil with the Atlantic Forest delimited (grey) following [18], forest remnants (red) following [66], and studied sites (black circles) for 132 species.

The Atlantic forest is a major global biodiversity hotspot [17] with a remaining forest area of only 11% of its original cover [18] (Figure 1). Unique climatic and geographic history provided heterogeneous environmental conditions for evolution of species and interactions leading to a high level of endemism in this biome. For instance, Orchidaceae has the highest species richness and endemism in the Brazilian Atlantic forest flora followed by Fabaceae, Asteraceae, and Bromeliaceae [19]. Despite considerable information on plant reproduction and pollination in this biome, synthetic studies of pollination sufficiency are lacking [20], but see [21]. In this descriptive review [21], PL was higher for species in the Orchidaceae and Fabaceae compared to other families but the patterns may be driven by phylogenetic non-independence in PL.

Given the extent of PL among angiosperms, previous review studies sought to identify factors (e.g., mating system and plant growth form) that explain its variation. Among them, pollinator specialization is expected to increase PL because specialist plants are more sensitive to stochasticity in pollination as a result of variation in pollinator abundance [22]–[23]. However, the term pollinator specialization refers to distinct concepts [24]–[26] that may relate to PL in different ways leading to conflicting results [27]. For instance, the number of pollinator species, or ecological specialization (after [26]), was positively related to the level of PL [4], [28], however, this association was not corroborated by studies that included visitation rates as measure of pollinator diversity [27], [29]. Phenotypic specialization (after [26]), on the other hand, predicts that complex floral architecture can limit pollinator access to floral rewards. Accordingly, pollinator richness was lower in zygomorphic than actinomorphic species, however, flower symmetry did not explain PL in a review of 26 angiosperms [27]. Nectar production may also indicate pollinator generalization, yet nectariferous species did not show lower PL than nectarless species, except when self-compatible species were excluded from the analysis [2]. Thus, tests of the relationship of PL and specialization on particular functional or taxonomic groups of pollinators (i.e., functional specialization after [26]) are needed, yet few studies have taken this approach [29].

Other life-history traits may be associated with PL. The most prominent relationship is between PL and self-incompatibility [1]–[4], [30]. For self-incompatible species, self-pollen fails to germinate or self-pollen tubes are arrested in the style due to the presence of genetic incompatibility systems [31]. Thus, self-incompatible species rely on pollinators to receive outcross pollen and achieve sexual reproduction. In contrast, the ability to self-fertilize in general decreases the likelihood of PL and the capacity for autogamy even more so (i.e., ability to set fruits after spontaneous self-fertilization) [2]–[3]. In fact, selfing ability is considered one adaptive response to chronic PL [32]. Plant lifetime may also negatively correlate with the occurrence of PL, as short-lived species may be less prone to PL than long-lived species. The reduction in seed production in one reproductive season may have a greater impact on lifetime fitness for short-lived species than long-lived ones who are able to compensate during other reproductive episodes. Thus, selection should favor traits that minimize PL in short-lived species. Indeed, woody species experience PL more often than herbs [2], [4]. However, this prediction was not upheld by the more general comparison of polycarpic *versus* monocarpic species [2], [4]. All together, the occurrence of PL among angiosperms has shown inconsistent associations with life-history traits tested thus far. It may be that this weak association results from combining studies across diverse biomes instead of considering species within a biome – which allow a comparative analysis at a large scale and in a relatively uniform abiotic and biotic environment (see also studies at community level [29], [33]–[34]).

In this study, we assessed the occurrence and magnitude of PL in large set of plants from a single biome, the Brazilian Atlantic forest. Furthermore, we tested if the phylogenetic relatedness affects the occurrence and extent of PL and whether pollination specialization, breeding system, and life habit explain variation in pollination sufficiency. Specifically, we sought to answer the questions:

1. Does PL increase with pollination specialization? For this, we consider three types of pollination specialization after [26]:
  - Ecological – Does PL decrease with number of pollinator species?
  - Phenotypic – Is PL higher in zygomorphic than actinomorphic species? And is PL lower in nectariferous than nectarless species?
  - Functional – Does PL decrease with number of pollinator functional groups? And does PL differ between species pollinated by invertebrates and vertebrates?
2. Is PL associated with plant breeding system?
  - Is PL higher in self-incompatible species than self-compatible?
  - Is PL higher in non-autogamous than autogamous species?
3. Does plant habit reflect higher PL in woody species than herbs?

## Materials and Methods

### Literature Review And Dataset

A review of published studies was conducted primarily using the databases ‘Institute for Scientific Information Web of Science’ and ‘Scientific Electronic Library Online – SciELO’. The following keyword combination (in Portuguese and English) was used: “reproductive biology” or “reproductive system” or “breeding system” or “mating system” or “self-incompatibility” or “pollination”. In the Web of Science the above terms were crossed with ‘Brazil’. Other papers, dissertations, and theses from personal library collections of the authors were added. Our search included papers published from 1985 through August 2012. The following criteria were used to select the studies: 1) conducted on native and bioticly pollinated species (only one study with one wind-pollinated species was found and it was not included); 2) conducted within the Atlantic forest domain (after [35] for Atlantic forest *sensu lato*, i.e., studies conducted within semi-deciduous and rain forests); 3) contained data on fruit set after hand cross-pollination and natural pollination (flowers exposed to pollinators). The following criteria were used to exclude species from the analysis: 1) species with fruit set after apomixis greater than 15%; 2) species with fruit set after natural pollination two-times (or more) greater than cross-pollination as these studies could reflect error induced by flower emasculation or flower manipulation. We used fruit set as the response variable because it is the most frequently reported measure in the pollination experiments located by our search (e.g., less than 10 studies reported seeds per flower or fruit) and fruit set can be a proxy of PL [36]–[37], but note that it comes with caveats (see [3], [38]).

**Figure 2:**
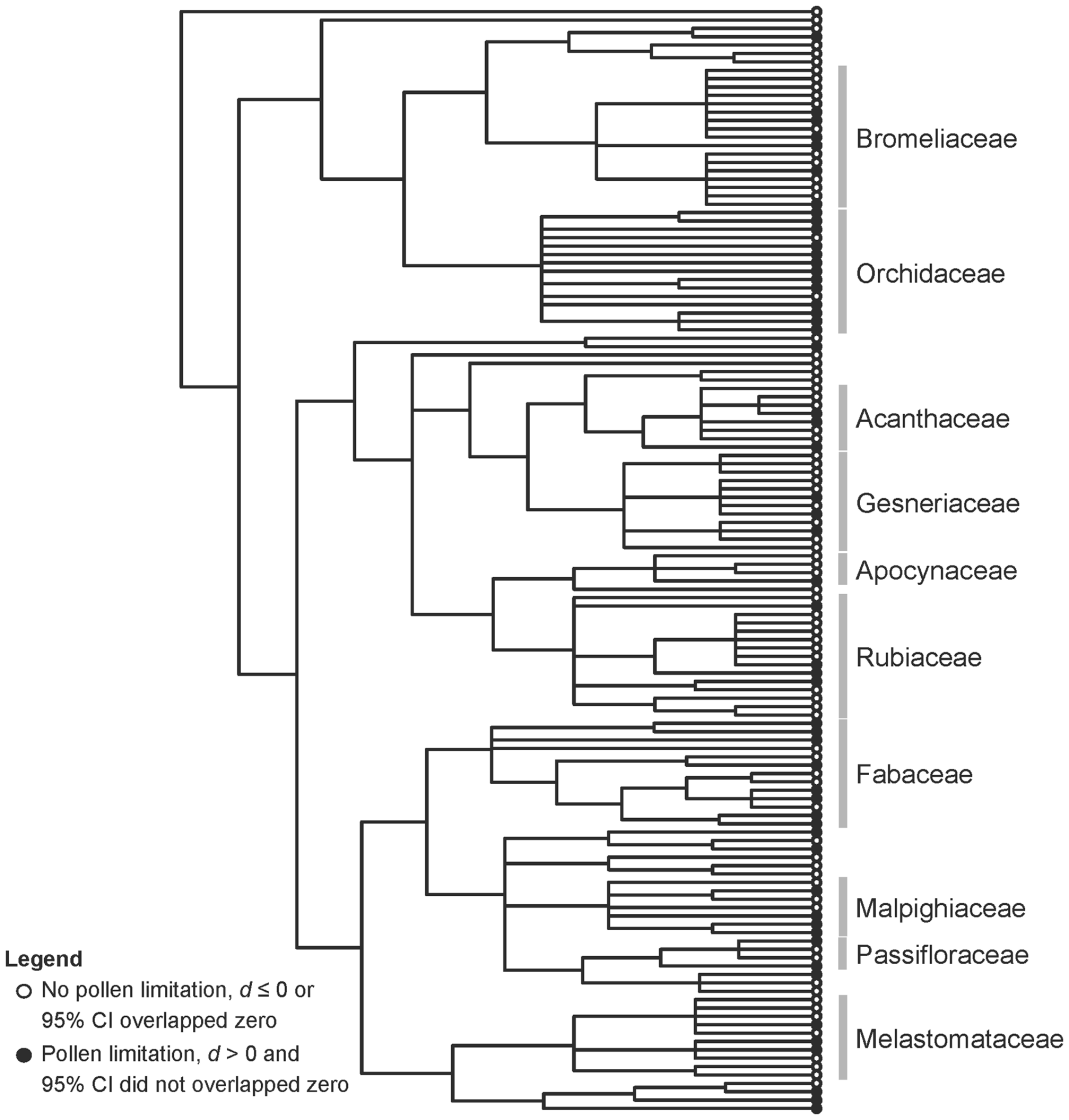
Distribution of pollen limitation across the phylogeny of plants in the Atlantic forest of Brazil. Occurrence of pollen limitation for the 132 species represented on the phylogeny obtained from the angiosperm APGIII [46] consensus tree (R20091110) in Phylomatic [47]. Pollen limitation was interpreted as significant if the effect size (*d*) was higher than zero and 95% confidence interval (CI) did not overlapped zero (black circles). Effect size (*d*) equal or lower than zero or 95% confidence interval (95% CI) that overlapped zero mean no pollen limitation (white circles).

In total our dataset was composed of 66 studies (one book chapter, 22 dissertations and theses, and 43 published papers) that performed hand cross- and natural pollination experiments in native and bioticly pollinated species within the Atlantic forest domain during the period 1985–2011. These studies quantified 173 records of 132 plant species (Table S1). Plant families Bromeliaceae (13% of species), Orchidaceae (11%), Rubiaceae (11%), and Fabaceae (10%) (Figure 2) had strong representation in the data set and most species (79%) were studied within the southeastern region of Brazil (Figure 1, Table S1). Species studied in more than one study or site (nine species) had an entry for each study/site and species with heteromorphic flowers (14 species) had an entry for each morph (but see DATA ANALYSIS). For all species, pollination experiments were performed at the partial-plant level (i.e., flowers, *sensu* [38]). For each entry we compiled data on number of flowers treated per treatment, number of developed and undeveloped fruits per treatment, and values of fruit set (treatments: autonomous self-pollination, hand self-pollination, hand cross-pollination and natural pollination) when available. We calculated species' compatibility system using the Index of Self-incompatibility (ISI, [39]), autogamy using the Index of Autogamy (IA, [40]), and the effect size of PL (see below) from these treatments. We also recorded other information regarding plant features used in the analyses (see in details below).

### Data Analysis

Pollen limitation of each species was estimated as the effect size based on the log odds ratio (ln(*o*)) obtained from 2×2 contingency table [41] of the numbers of developed and undeveloped fruits of cross- and natural pollination. The variance of ln(*o*) (V_ln(*o*)_) was estimated as the sum of inverses of the number of developed and undeveloped fruits of cross- and natural pollination. The ln(*o*) and V_ln(*o*)_ were transformed to *d* (standardized mean difference) and variance of *d* respectively, to facilitate interpretation and comparison with other studies (cf. [41] for detailed information on the calculations and equations). For species with more than one entry we selected the study/site with the lower variance value to designate the effect size for that species. For species with heteromorphic flowers, we calculated the species' effect size from the sum of the number of developed and undeveloped fruits per treatment per morph. For a given species (or overall), PL was interpreted as significant when the effect size was positive and its 95% confidence intervals did not overlap zero [42]. Negative values of effect size or 95% confidence intervals that overlapped zero represent no PL.

Overall effect size was estimated by random-effects models, which take in to account the deviation from the true effect size that may be generated by differences between the studies (e.g., sample size) [41]. Model assumptions and publication bias were analyzed by a variety of methods (normal Q-Q plot, influence plot, funnel plot, symmetry test, and Rosenberg fail-safe number) with the metafor package [43] in R.15.0 [44]. The normal Q-Q plot was used to evaluate normality (i.e., points should fall within the confidence bands) whereas the influence plot can indicate the presence of outliers in the dataset (e.g., studies with large residuals are showed as red points). Funnel plot, symmetry test, and Rosenberg fail-safe number are complementary methods and were used to identify and examine the potential impact of publication bias in meta-analysis. Because Rosenberg fail-safe number (18690) was much larger than the critical value (660), there was no evidence of publication bias in the dataset. Specifically, fail-safe number indicates the number of non-significant, unpublished, or missing studies that would need to exist to overturn the results [41]. However, visual inspection of normal Q-Q plot, funnel plot, and influence plot, and the symmetry test (t = 2.08, *df* = 130, *P* = 0.04) for 132 species indicated six outliers in the dataset (Figure S1). For the subset without outliers, normality and symmetry (t = 0.90, *df* = 124, *P* = 0.37) were acceptable (Figure S1), thus, we conducted the following meta-analyses with this subset of 126 species.

We calculated overall effect size by traditional and phylogenetically independent meta-analyses in Phylometa 1.3 beta [45]. Traditional and phylogenetically independent meta-analyses had comparable Akaike's information criterion (AIC) values (absolute difference lower than 5 units, Table S2), thus we focus our description and interpretation of results on the phylogenetically independent random-effects models.

The phylogenetic hypothesis was obtained using the angiosperm APG III [46] consensus tree (R20091110) from Phylomatic [47]. Branch lengths were calibrated from the minimum age of clade divergence [48] using the branch length adjuster function (BLADJ) from Phylocom [49].

The effect of categorical plant features on PL was tested in Phylometa. We conducted one meta-analysis for each categorical plant feature because the number of species with data on a given plant feature differed among them. Within each plant feature, a category was included in the analysis if number of species for a given level of that category was equal or greater than eight. The following eight features (with two or more levels) were tested: flower symmetry (actinomorphic, zygomorphic); floral reward (nectariferous [nectar or nectar and others rewards], nectarless [others rewards]); number of pollinator functional groups (1, 2, ≥3 from the following functional groups: bat, bird, bee, beetle, butterfly, hawkmoth, hoverfly, other fly, moth, wasp, other); pollinator group (vertebrate, invertebrate, mixed); number of pollinator species (1, 2–5, >5, following [4]); mating system (self-compatible [ISI ≥0.30], self-incompatible [ISI <0.30], following [50]); autogamy (autogamous [IA ≥0.30], non-autogamous [IA <0.30], following [50]); and plant habit (herbs, woody plants [shrubs, treelets, trees], vines).

## Results

### Occurrence And Magnitude Of Pollen Limitation

The magnitude of PL was moderate, 0.50, and significantly different from zero, 95% confidence interval [0.37, 0.62], for plants in the Brazilian Atlantic forest. PL was observed in 39% (49) of species (Figure 2, Table S1). The overall heterogeneity of effect size was large and statically significant (Q = 727.83, *df* = 25, *P*<0.001), indicating that PL varied among species and the influence of plant features was warranted (see below).

### Pollen Limitation And Association With Plant Features

**Figure 3:**
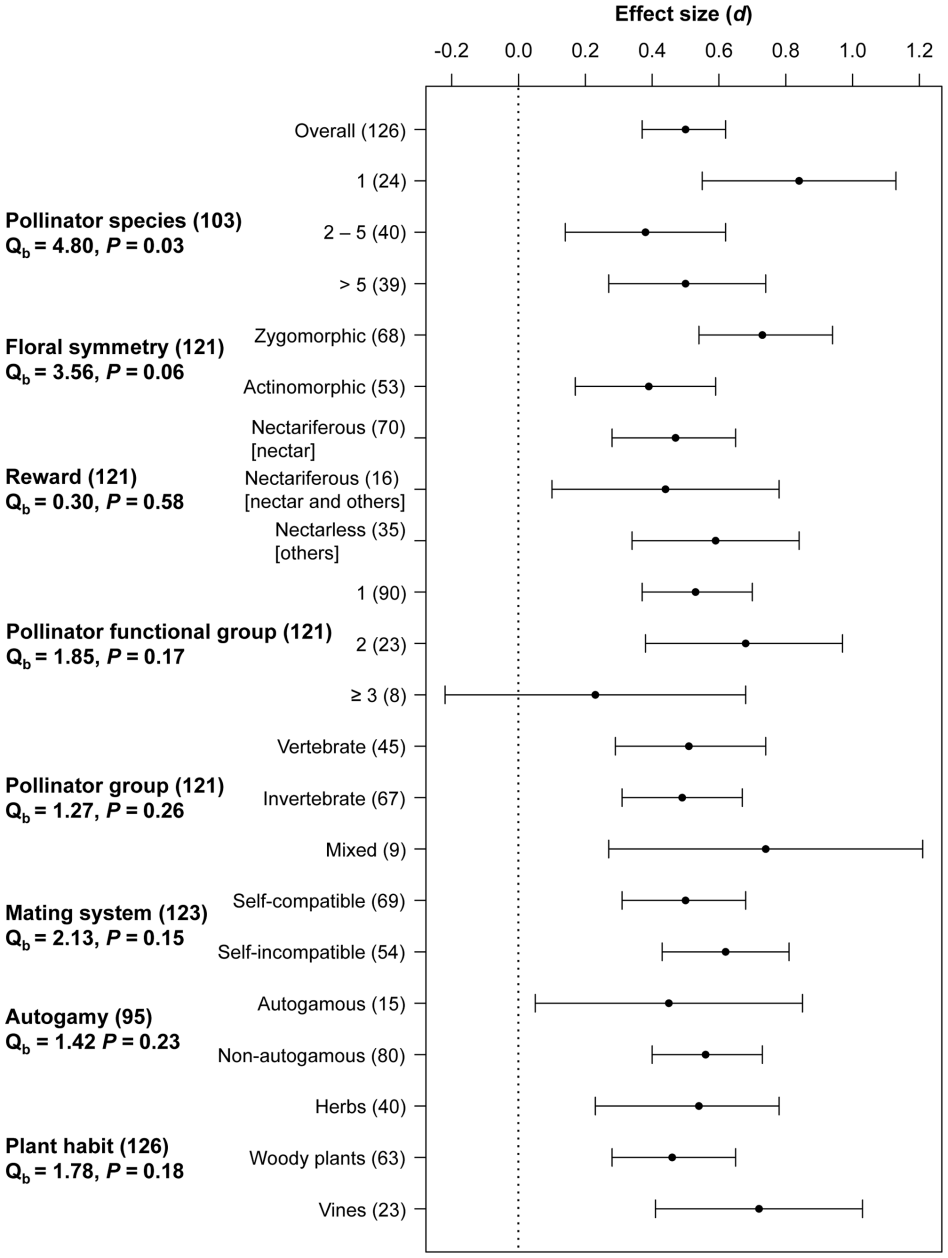
Effect size and 95% confidence interval of pollen limitation in the Atlantic forest of Brazil. Overall effect size (*d*) and 95% confidence interval of pollen limitation and per category based on phylogenetically independent meta-analysis. Heterogeneity between categories of plant features (Q_b_) and *P* value (N = number of species per category) are presented.

The association of PL with plant features was evaluated by phylogenetically independent meta-analysis to avoid interpretation bias due to phylogenetic relatedness among species (Figure 3, Table S2). The heterogeneity among groups was significant for ecological pollination specialization (i.e., number of pollinator species), and marginally significant for phenotypic pollination specialization (exclusively for floral symmetry) (Figure 3). This means that extreme ecological specialists (plants pollinated by one species) had higher PL than more generalists (two or more species of pollinators) and zygomorphic species had higher PL than actinomorphic species. However, PL was not lower in nectariferous than nectarless species. Even though heterogeneity was not significant for functional pollination specialization, functional generalist plants pollinated by three or more pollinator functional groups were not pollen limited (Figure 3). Heterogeneity among groups was not significant for other plant features, i.e., breeding system and life habit. However PL was significant for most categories within each plant feature (e.g., both self-compatible and self-incompatible species were pollen limited) (Figure 3).

## Discussion

Pollen limitation was frequently observed among plants of the Brazilian Atlantic forest. The magnitude of PL was moderate (0.50) and comparable to previous meta-analytical studies (0.52 [4]), although the proportion (39%) of species with PL was lower than the 62–73% recorded for angiosperms as a whole [1], [3]. While overestimation of PL may be due to publication bias, experimental design, or the response variable measured [38], none seem to be influential here. First, publication bias was not found in this study, possibly due to inclusion of theses and dissertations in the dataset. Second, while whole-plant level experiments estimate higher levels of PL than partial-plant level experiments [38], as a result of resource reallocation among flowers and inflorescences [51]–[53], in this review, PL was estimated from fruit set at the partial-plant level (i.e., flowers), which does not differ from whole-plant level estimation across angiosperms [38]. Lastly, even though fruit set may estimate greater magnitude of PL than seed set measures [38], both response variables are correlated [2], [38] and fruit set is the most reported response variable both in previous meta-analyses and the studies included in this review. The magnitude of PL can increase when quality measures are included [29], [32], [38], [54]–[55]. Although both quantity and quality components are complementary measures of PL [29], [32], [38], [54], only quantity variables have been widely included in review studies thus far.

Pollinator specialization had the strongest effect on PL effect size in the Atlantic forest. But of the three approaches of pollinator specialization [26] explored only two were significant indicators of PL. Ecological specialists (i.e., plants with one pollinator species) had higher levels of PL than generalist species pollinated by two or more species corroborating other studies [4], [56]. Likewise, phenotypic specialization measured by floral symmetry, showed that specialist (zygomorphic) species experienced marginally more PL than generalist (actinomorphic) species. These results suggest that plants with zygomorphic flowers may depend upon precise pollen deposition [57]–[58], but when pollinators with the right ‘fit’ are a minor part of the assemblage other visitors pollinate these plants. Plants with zygomorphic flowers, thus, may be more prone to the effects of heterogeneous pollination environments. Lastly, although functional specialization did not significantly explain variation in PL, functional generalization alleviated PL when number of pollinator functional groups was high. Vertebrate and invertebrate pollinators were equally effective and the extent of PL was similar between nectariferous and nectarless species – results that differ from our expectations but corroborates results of reviews across angiosperms [2].

Phenotypic and ecological specializations lead to high levels of PL under a scenario of pollination decay or unpredictability [59]–[60]. Under this scenario, generalist species will be more competitive when reproductive success is achieved by replacement of pollinator species with similar pollination efficiency. Hence generalist pollination systems are more resistant to fluctuations in pollination service [61]. Thus, our findings plus the fact that tropical plants may have relatively more specialized pollination systems than temperate ones [24] suggest that plants which depend on a single pollinator species in this biome may be most prone to the reproductive failure as a result of pollinator loss.

Deviating from expectation [2], [4], [27], self-compatible species were not less prone to PL than self-incompatible species in the Atlantic forest. This may result from the fact that most (84%) of the species (71% of the self-compatible ones) in this review were non-autogamous system (i.e., have a low ability to set fruit after spontaneous self-fertilization). Thus, species in the Brazilian Atlantic forest depend upon pollinators to achieve reproductive success regardless of breeding system.

Lack of association of PL and life habit was also reported [4]. Here this may reflect herbaceous species not being as short-lived plants as traditionally thought. For instance species in Bromeliaceae and Orchidaceae while not woody do exhibit clonal propagation [62] and thus may be long-lived and have many opportunities for sexual reproduction. Thus, PL in a given season may not significantly affect fitness across the whole lifespan. Studies assessing the occurrence of PL over time and in non-woody clonal plants would be needed to separate the effects of woodiness and longevity.

## Conclusions

Pollen limitation was observed in the Brazilian Atlantic forest and its magnitude was comparable with angiosperms as a whole. Despite the large dataset analyzed here, it represents approximately only 1% of the plant richness in the Atlantic forest [19]. Indeed, tropical regions are the areas with highest species richness and where the fewest pollen supplementation studies have been conducted [12]. Moreover, our knowledge is concentrated in southeastern Brazil, so reproductive and pollination studies or species in the richest sites across the Atlantic forest (e.g., Bahia and Espirito Santo states [63]–[64]) are still needed. Furthermore, we strongly recommend that future studies assess both quantity and quality components of PL, and especially pre-zygotic measures to avoid bias due to resource reallocation [32], [54].

Pollination specialization was the most powerful predictor of PL among plants in the Brazilian Atlantic forest. Although other plant traits (breeding system and life habit) did not affect PL in this review, analysis that includes the interaction between factors could facilitate deeper understanding of the determinants of PL [13]. Nevertheless, results presented here indicate a stochastic component in pollen receipt [13] so it will be important for future studies of PL in the Atlantic forest to consider the evolution of excess ovules [65] in the context of pollination specialization.

## Supporting Information

Figure S1 **Diagnostic of random-effects models.** Diagnostic for model assumptions and publication bias: normal Q-Q plot, funnel plot, influence plot, symmetry test, overall effect size, heterogeneity, Rosenberg fail-safe number, and critical value for the dataset with (132 species) and without outliers (126).
(PDF)

Table S1 **Data, location (longitude and latitude), and reference for the studies included in this review.** Percentage of fruit set after hand cross-pollination (CP) and natural pollination (NP), effect size (*d*), variance (*v*), and 95% confidence interval (95% CI) for 132 species in the Atlantic forest of Brazil.
(PDF)

Table S2 **Results of traditional and phylogenetically independent meta-analyses based on random-effects models.** Heterogeneity between categories (Q_b_), degrees of freedom (*df*), *P* value, and Akaike's information criterion (AIC) per plant feature. Effect size (*d*) and 95% confidence interval (95% CI), Z value, degrees of freedom (*df*), and *P* value per category of plant feature.
(PDF)
