# Supplementary material for: Meta-Analysis of Pollen Limitation Reveals the Relevance of Pollination Generalization in the Atlantic Forest of Brazil
Source: PLoS One. 2014 Feb 21;9(2):e89498. doi: 10.1371/journal.pone.0089498 (PMC3931788; doi:10.1371/journal.pone.0089498)

Figure S1. Diagnostic of random-effects models. Diagnostic for model assumptions and publication bias: normal Q-Q plot, funnel plot, influence plot, symmetry test, overall effect size, heterogeneity, Rosenberg fail-safe number, and critical value for the dataset with (132 species) and without outliers (126).

Normal Q-Q plot 132 species

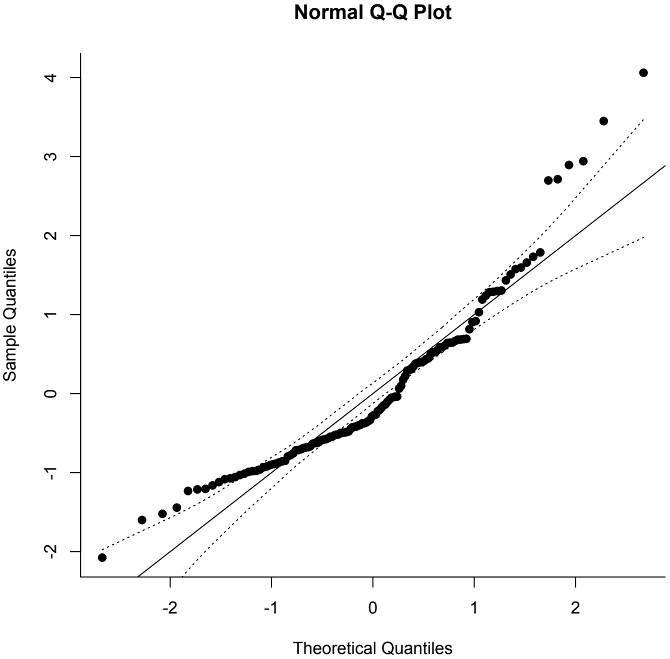

126 species

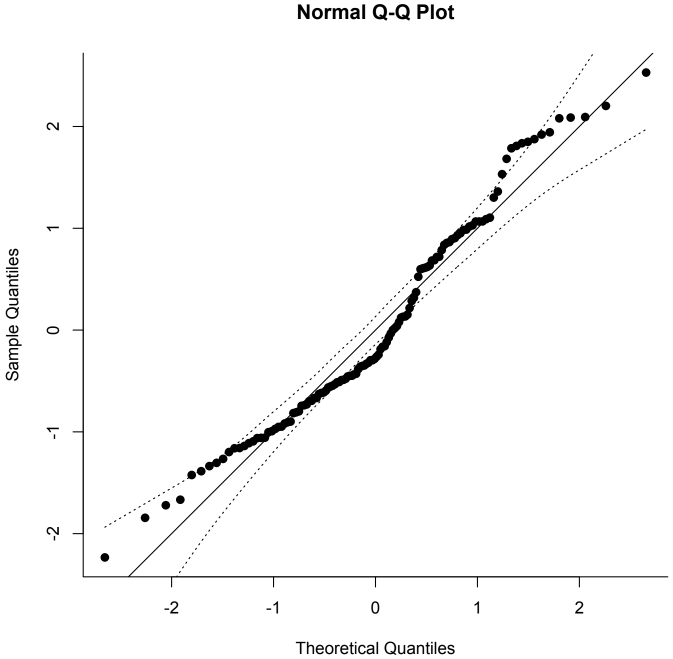

Funnel plot 132 species

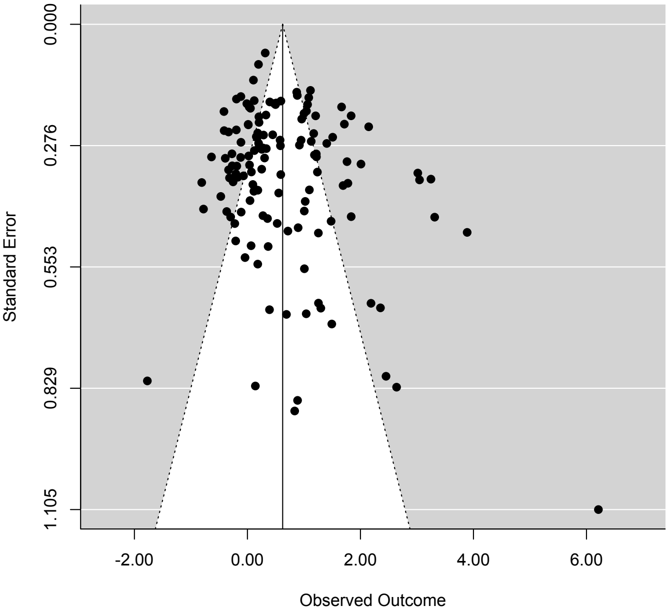

126 species

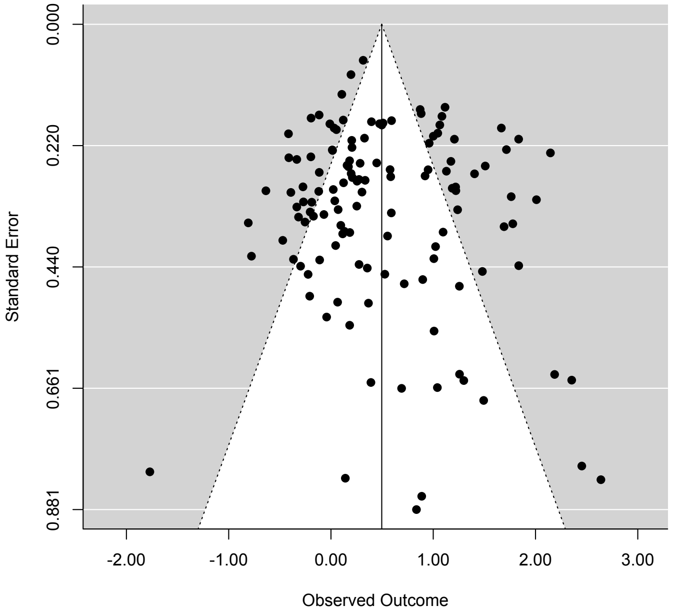

symmetry test: t = 2.08, *df* = 130, *P* = 0.04
overall effect size 0.62 [0.47, 0.77]
heterogeneity: Q = 1015.96, *df* = 131, *P* < 0.001

Rosenberg fail-safe number: 18690
critical value = 660

symmetry test: t = 0.90, *df* = 124, *P* = 0.37
overall effect size 0.50 [0.37, 0.62]
heterogeneity: Q = 727.83, *df* = 125, *P* < 0.001

Rosenberg fail-safe number: 14382
critical value = 640

Figure S1. Continued.

Influence plot 132 species

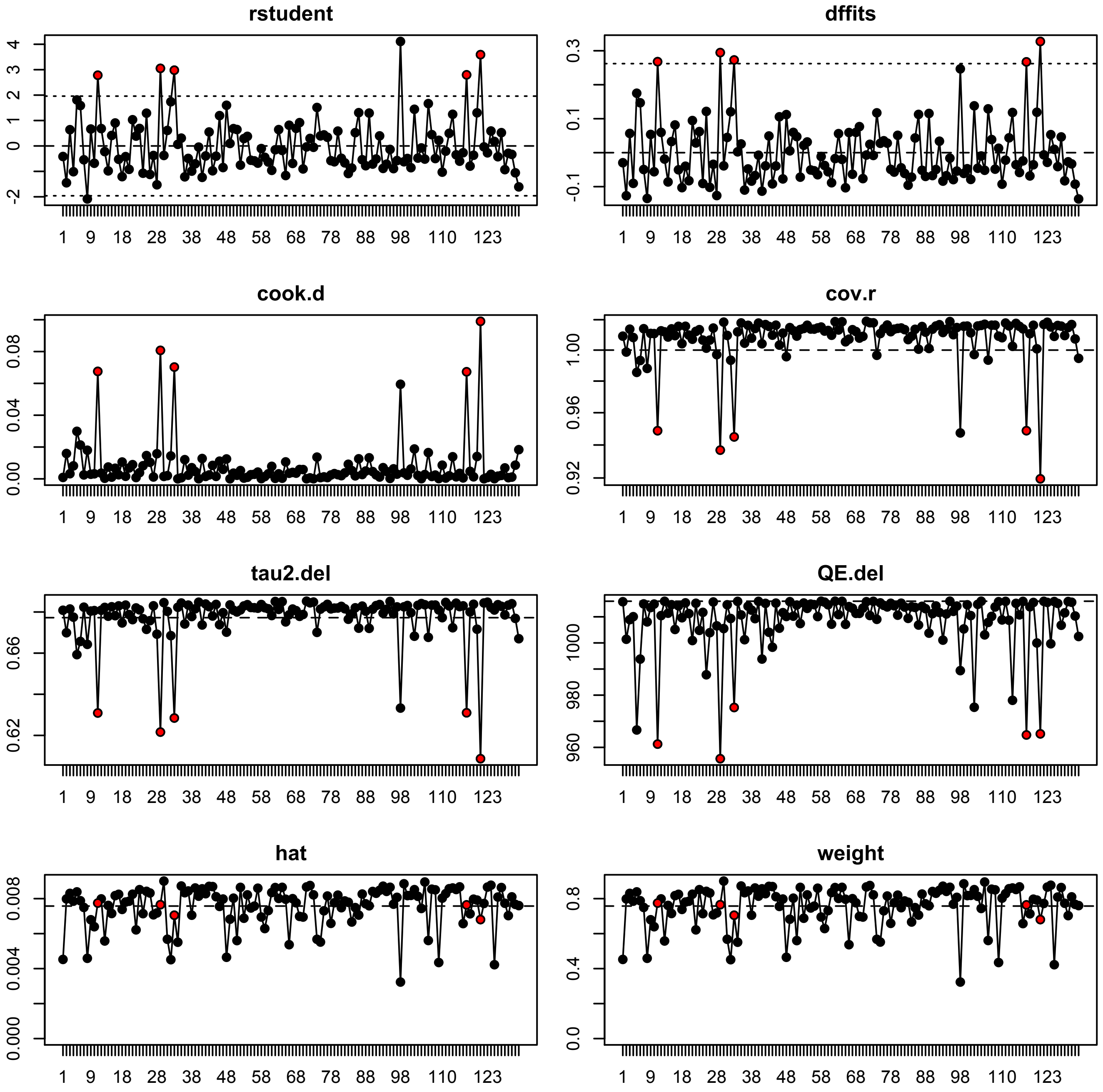

Figure S1. Continued.

Influence plot 126 species

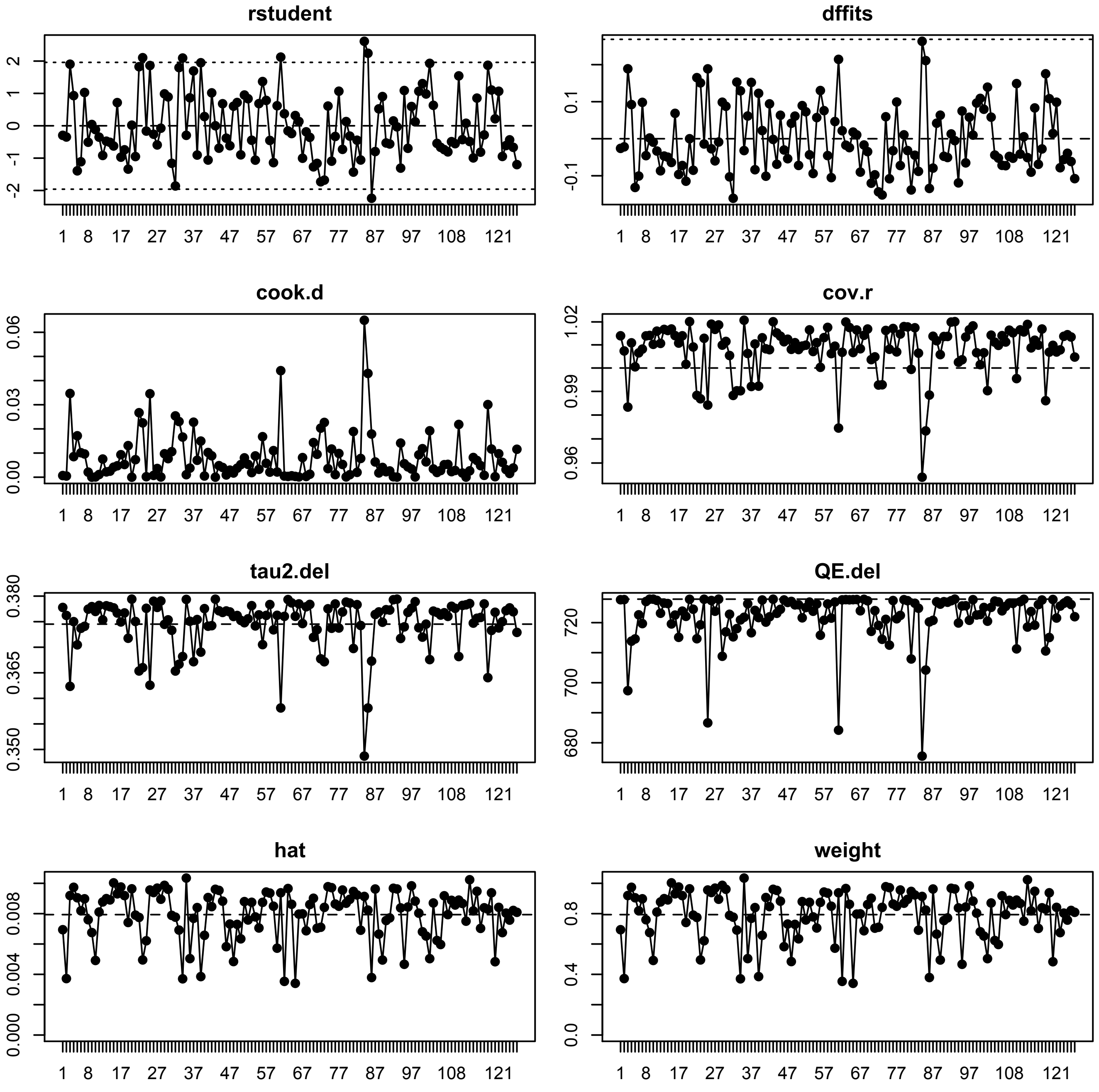

Supplement: Figure S1 — Diagnostic of random-effects models. Diagnostic for model assumptions and publication bias: normal Q-Q plot, funnel plot, influence plot, symmetry test, overall effect size, heterogeneity, Rosenberg fail-safe number, and critical value for the dataset with (132 species) and without outliers (126). (PDF) [file pone.0089498.s001.pdf]
