# Supplementary material for: Meta-Analysis of Pollen Limitation Reveals the Relevance of Pollination Generalization in the Atlantic Forest of Brazil
Source: PLoS One. 2014 Feb 21;9(2):e89498. doi: 10.1371/journal.pone.0089498 (PMC3931788; doi:10.1371/journal.pone.0089498)
Supplement: Table S1 — Data, location (longitude and latitude), and reference for the studies included in this review. Percentage of fruit set after hand cross-pollination (CP) and natural pollination (NP), effect size (d), variance (v), and 95% confidence interval (95% CI) for 132 species in the Atlantic forest of Brazil. (PDF) [file pone.0089498.s002.pdf]

Table S1. Data, location (longitude and latitude), and reference for the studies included in this review. Percentage of fruit set after hand cross-pollination (CP) and natural pollination (NP), effect size (*d*), variance (*v*), and 95% confidence interval (95% CI) for 132 species in the Atlantic forest of Brazil.

Family	Species	CP	NP	*d*	*v*	95% CI	Longitude	Latitude	Reference
Acanthaceae	*Geissomeria perbracteosa*	61.54	70.00	-0.21	0.08	-1.18, 0.76	-46.933333	-22.841667	Pereira 1998
Acanthaceae	*Justicia sebastianopolitana*	33.33	32.10	0.03	0.05	-0.34, 0.40	-44.600000	-22.450000	Wolowski 2013
Acanthaceae	*Odontonema barbelerioides*	27.78	11.76	0.58	0.06	0.04, 1.13	-44.600000	-22.450000	Wolowski 2013
Acanthaceae	*Ruellia brevifolia*	90.32	56.10	1.10	0.12	0.36, 1.84	-49.116667	-22.783333	Sigrist and Sazima 2002
Acanthaceae	*Ruellia menthoides*	100.00	66.67	0.89	0.03	-0.79, 2.57	-42.858611	-20.801944	Lima and Vieira 2006
Acanthaceae	*Ruellia subsessilis*	85.71	62.07	0.72	0.13	-0.21, 1.64	-42.858611	-20.801944	Lima and Vieira 2006
Acanthaceae	*Staurogyne itatiaiae*	27.27	44.34	-0.42	0.28	-0.80, -0.03	-44.600000	-22.450000	Wolowski 2013
Alismataceae	*Echinodorus longipetalus*	98.33	90.46	1.01	0.03	-0.08, 2.10	-48.498056	-21.745556	Pansarin 2008a
Annonaceae	*Anaxagorea dolichocarpa*	22.22	15.31	0.25	0.03	-0.39, 0.90	-34.990278	-7.798056	Braun 2010
Apocynaceae	*Oxypetalum mexiae*	10.71	0.49	0.13	0.40	-0.60, 0.87	-42.875000	-20.750000	Vieira and Grabalos 2003
Apocynaceae	*Prestonia coalita*	66.67	21.88	3.31	0.18	2.45, 4.17	-47.116667	-22.833333	Franco 1991
Apocynaceae	*Prestonia riedelli*	16.22	6.98	-0.20	0.09	-0.87, 0.46	-47.116667	-22.833333	Franco 1991
Apocynaceae	*Rauvolfia grandiflora*	12.50	8.63	0.36	0.12	-0.51, 1.22	-34.875000	-8.125000	Lopes and Machado 1999
Boraginaceae	*Cordia multispicata*	52.73	47.92	0.11	0.18	-0.14, 0.36	-34.875000	-8.125000	Machado and Loiola 2000
Bromeliaceae	*Aechmea nudicaulis*	62.07	35.85	0.59	0.03	0.25, 0.94	-44.600000	-22.450000	Wolowski 2013
Bromeliaceae	*Aechmea vanhoutteana*	91.67	94.00	-0.19	0.07	-0.53, 0.14	-44.600000	-22.450000	Wolowski 2013
Bromeliaceae	*Araeococcus parviflorus*	30.00	23.05	0.20	0.09	-0.33, 0.73	-35.841667	-8.711667	Siqueira Filho 2003

Table S1. Continued.

Family	Species	CP	NP	*d*	*v*	95% CI	Longitude	Latitude	Reference
Bromeliaceae	*Billbergia distachia*	86.96	80.22	0.27	0.05	-0.28, 0.83	-44.600000	-22.450000	Wolowski 2013
Bromeliaceae	*Nidularium itatiaiae*	82.05	48.45	0.87	0.03	0.57, 1.18	-44.600000	-22.450000	Wolowski 2013
Bromeliaceae	*Pitcairnia flammea*	92.68	81.62	0.58	0.04	0.06, 1.09	-44.600000	-22.450000	Wolowski 2013
Bromeliaceae	*Quesnelia augusto-coburgii*	60.32	60.78	-0.01	0.05	-0.36, 0.34	-44.600000	-22.450000	Wolowski 2013
Bromeliaceae	*Quesnelia lateralis*	80.00	44.00	0.90	0.13	-0.01, 1.81	-42.516667	-22.408333	Martinelli 1997
Bromeliaceae	*Tillandsia geminiflora*	95.65	29.41	2.19	0.03	0.94, 3.43	-44.600000	-22.450000	Wolowski 2013
Bromeliaceae	*Vriesea ensiformis*	70.00	26.74	1.02	0.31	0.23, 1.81	-47.250000	-24.500000	Araujo et al. 1994
Bromeliaceae	*Vriesea carinata*	75.00	31.25	1.04	0.11	-0.25, 2.33	-47.250000	-24.500000	Araujo et al. 1994
Bromeliaceae	*Vriesea gradata*	36.36	9.72	0.92	0.07	0.38, 1.46	-44.600000	-22.450000	Wolowski 2013
Bromeliaceae	*Vriesea heterostachys*	66.67	64.00	0.06	0.16	-0.92, 1.05	-42.516667	-22.408333	Martinelli 1997
Bromeliaceae	*Vriesea hydrophora*	50.00	55.00	-0.11	0.68	-0.95, 0.73	-42.516667	-22.408333	Martinelli 1997
Bromeliaceae	*Vriesea longicaulis*	50.00	36.59	0.30	0.15	-0.29, 0.90	-44.600000	-22.450000	Wolowski 2013
Bromeliaceae	*Vriesea longiscapa*	60.00	86.00	-0.78	0.26	-1.60, 0.05	-42.516667	-22.408333	Martinelli 1997
Bromeliaceae	*Vriesea sparsiflora*	57.14	54.00	0.07	0.09	-0.59, 0.73	-42.516667	-22.408333	Martinelli 1997
Campanulaceae	*Lobelia thapsoidea*	76.32	100.00	-1.77	0.09	-3.36, -0.18	-42.658333	-22.275000	Mello 2004
Campanulaceae	*Siphocampylus humilis*	86.67	40.00	1.26	0.11	0.32, 2.19	-42.658333	-22.275000	Mello 2004
Clusiaceae	*Clusia nemorosa*	88.61	85.39	0.16	0.04	-0.34, 0.66	-34.875000	-8.125000	Lopes and Machado 1998
Clusiaceae	*Vismia guianensis*	60.00	40.00	0.45	0.05	-0.05, 0.94	-34.875000	-8.125000	Lopes and Machado 1998
Commelinaceae	*Dichorisandra hexandra*	65.63	18.18	1.19	0.13	0.60, 1.77	-47.116667	-22.816667	Sigrist 1992
Commelinaceae	*Dichorisandra incurva*	31.82	43.40	-0.27	0.07	-0.85, 0.30	-47.116667	-22.816667	Sigrist 1992
Euphorbiaceae	*Croton floribundus*	73.08	30.65	1.00	0.09	0.60, 1.40	-47.109167	-22.829167	Passos 1995
Euphorbiaceae	*Croton priscus*	35.00	27.14	0.20	0.64	-0.21, 0.62	-47.109167	-22.829167	Passos 1995

Table S1. Continued.

Family	Species	CP	NP	*d*	*v*	95% CI	Longitude	Latitude	Reference
Euphorbiaceae	*Dalechampia stipulacea*	100.00	33.33	2.64	0.10	1.02, 4.26	-47.100000	-23.816667	Sazima and Sazima 1985
Fabaceae	*Caesalpinia echinata*	41.03	9.17	1.06	0.02	0.71, 1.42	-35.194444	-8.039722	Borges et al. 2009
Fabaceae	*Canavalia picta*	32.56	40.82	-0.20	0.18	-0.67, 0.27	-47.109167	-22.829167	Franco 1995
Fabaceae	*Dioclea rufescens*	36.54	18.95	0.50	0.11	0.14, 0.86	-47.109167	-22.829167	Franco 1995
Fabaceae	*Inga ingoides*	10.00	14.97	-0.25	0.21	-0.96, 0.45	-35.866667	-9.000000	Cruz Neto 2007
Fabaceae	*Inga striata*	6.67	1.14	1.01	0.40	0.17, 1.84	-35.866667	-9.000000	Cruz Neto 2007
Fabaceae	*Inga vera*	20.00	1.92	1.40	0.16	0.87, 1.94	-35.866667	-9.000000	Cruz Neto 2007
Fabaceae	*Mucuna japira*	36.36	2.71	1.67	0.04	1.30, 2.04	-44.833333	-23.350000	Agostini 2008
Fabaceae	*Mucuna urens*	73.53	11.01	1.72	0.08	1.27, 2.16	-44.833333	-23.350000	Agostini 2008
Fabaceae	*Pseudopiptadenia contorta*	22.73	25.00	-0.07	0.07	-0.75, 0.61	-42.283333	-22.525000	Pires and Freitas 2008
Fabaceae	*Pseudopiptadenia leptostachya*	16.67	28.00	-0.37	0.12	-1.20, 0.47	-42.283333	-22.525000	Pires and Freitas 2008
Fabaceae	*Senna multijuga*	52.88	14.44	1.04	0.06	0.66, 1.43	-44.600000	-22.450000	Wolowski and Freitas 2010
Fabaceae	*Swartzia pickelii*	5.88	0.25	1.78	0.07	1.07, 2.49	-34.875000	-8.125000	Lopes and Machado 1996
Fabaceae	*Vigna candida*	70.00	68.24	0.05	0.66	-0.74, 0.83	-47.109167	-22.829167	Franco 1995
Gentianaceae	*Irlbachia alata*	100.00	93.94	0.14	0.04	-1.47, 1.76	-34.933333	-8.016667	Machado et al. 1998
Gesneriaceae	*Besleria longimucronata*	45.45	30.00	0.37	0.00	-0.63, 1.36	-44.833333	-23.366667	San-Martin Gajardo and Freitas 1999
Gesneriaceae	*Nematanthus crassifolius*	57.89	73.68	-0.39	0.03	-0.99, 0.21	-44.600000	-22.450000	Wolowski 2013
Gesneriaceae	*Nematanthus fornix*	75.00	16.67	1.49	0.12	0.15, 2.83	-44.600000	-22.450000	Wolowski 2013
Gesneriaceae	*Nematanthus lanceolatus*	45.45	44.44	0.02	0.04	-0.57, 0.61	-44.600000	-22.450000	Wolowski 2013

Table S1. Continued.

Family	Species	CP	NP	*d*	*v*	95% CI	Longitude	Latitude	Reference
Gesneriaceae	*Sinningia cooperi*	87.50	42.55	1.24	0.07	0.58, 1.90	-44.600000	-22.450000	Wolowski 2013
Gesneriaceae	*Sinningia eumorpha*	61.54	69.23	-0.19	0.14	-0.82, 0.44	-47.933333	-22.400000	San-Martin Gajardo 2004
Gesneriaceae	*Sinningia gigantifolia*	57.14	8.33	1.48	0.12	0.60, 2.36	-44.600000	-22.450000	Wolowski 2013
Gesneriaceae	*Sinningia schiffneri*	50.00	44.44	0.12	0.13	-0.44, 0.69	-46.321944	-23.778056	San-Martin Gajardo 2004
Gesneriaceae	*Sinningia tuberosa*	47.06	38.89	0.18	0.01	-0.56, 0.92	-43.883333	-21.700000	San-Martin Gajardo 2004
Gesneriaceae	*Vanhouttea brueggeri*	45.00	43.33	0.04	0.05	-0.59, 0.67	-43.883333	-21.700000	San-Martin Gajardo 2004
Gesneriaceae	*Vanhouttea calcarata*	50.00	60.00	-0.22	0.19	-1.11, 0.67	-43.016667	-22.450000	San-Martin Gajardo 2004
Gesneriaceae	*Vanhouttea hilariana*	52.17	66.67	-0.33	0.42	-0.98, 0.32	-43.883333	-21.700000	San-Martin Gajardo 2004
Lamiaceae	*Salvia sellowiana*	51.72	38.98	0.29	0.19	-0.21, 0.78	-44.600000	-22.450000	Wolowski 2013
Malpighiaceae	*Banisteriopsis adenopoda*	16.67	8.89	0.40	0.22	0.05, 0.74	-47.116667	-22.816667	Sigrist and Sazima 2004
Malpighiaceae	*Banisteriopsis muricata*	38.10	2.78	1.69	0.11	0.97, 2.41	-47.116667	-22.816667	Sigrist and Sazima 2004
Malpighiaceae	*Byrsonima sericea*	47.95	10.86	1.12	0.19	0.82, 1.41	-34.875000	-8.125000	Teixeira and Machado 2000
Malpighiaceae	*Dicella bracteosa*	5.26	0.00	0.84	0.08	-0.89, 2.56	-47.116667	-22.816667	Sigrist and Sazima 2004
Malpighiaceae	*Mascagnia anisopetala*	3.28	4.04	-0.12	0.03	-0.71, 0.47	-47.116667	-22.816667	Sigrist and Sazima 2004

Table S1. Continued.

Family	Species	CP	NP	*d*	*v*	95% CI	Longitude	Latitude	Reference
Malpighiaceae	*Mascagnia cordifolia*	42.86	0.00	2.45	0.09	0.88, 4.02	-47.116667	-22.816667	Sigrist and Sazima 2004
Malpighiaceae	*Stigmaphyllon lalandianum*	7.62	4.94	0.25	0.05	-0.30, 0.81	-47.116667	-22.816667	Sigrist and Sazima 2004
Malvaceae	*Abutilon bedfordianum*	72.41	34.64	0.88	1.22	0.56, 1.20	-44.600000	-22.450000	Wolowski 2013
Marantaceae	*Calathea cylindrica*	35.14	40.00	-0.11	0.19	-0.64, 0.41	-43.216667	-22.966667	Barreto and Freitas 2007
Marantaceae	*Calathea villosa*	20.00	30.77	-0.32	0.42	-1.00, 0.37	-35.183333	-8.016667	Teixeira 2005
Marantaceae	*Hylaeanthe hexantha*	60.00	86.67	-0.81	0.21	-1.51, -0.10	-35.183333	-8.016667	Teixeira 2005
Melastomataceae	*Leandra dasytricha*	55.17	45.71	0.21	0.09	-0.34, 0.75	-46.983333	-23.266667	Goldenberg and Varassin 2001
Melastomataceae	*Leandra regnellii*	85.71	68.75	0.55	0.09	-0.20, 1.31	-46.983333	-23.266667	Goldenberg and Varassin 2001
Melastomataceae	*Miconia ciliata*	60.00	76.00	-0.41	0.10	-0.89, 0.06	-34.875000	-8.062500	Melo and Machado 1998
Melastomataceae	*Miconia elegans*	43.28	30.12	0.31	0.14	0.19, 0.44	-47.116667	-22.833333	Borges 1991
Melastomataceae	*Miconia pusilliflora*	20.16	15.04	0.20	0.04	0.02, 0.37	-46.983333	-23.266667	Goldenberg and Varassin 2001
Melastomataceae	*Tibouchina cerastifolia*	67.39	58.73	0.21	0.23	-0.23, 0.64	-48.987778	-25.433889	Franco 2007
Melastomataceae	*Tibouchina clinopodifolia*	96.67	73.33	1.30	0.07	0.03, 2.57	-48.987778	-25.433889	Franco 2007
Melastomataceae	*Tibouchina gracilis*	89.74	93.75	-0.30	0.06	-1.16, 0.56	-48.987778	-25.433889	Franco 2007
Melastomataceae	*Tibouchina sellowiana*	80.00	76.47	0.11	0.04	-0.63, 0.86	-46.983333	-23.266667	Goldenberg and Varassin 2001
Melastomataceae	*Tibouchina semidecandra*	62.50	58.33	0.10	0.68	-0.62, 0.81	-46.983333	-23.266667	Goldenberg and Varassin 2001

Table S1. Continued.

Family	Species	CP	NP	*d*	*v*	95% CI	Longitude	Latitude	Reference
Orchidaceae	*Acianthera aveniformis*	35.00	2.16	1.76	0.43	1.15, 2.38	-49.234722	-25.559722	Dos Santos Filho 2007
Orchidaceae	*Acianthera hygrophila*	60.00	0.41	3.25	0.08	2.56, 3.94	-49.234722	-25.559722	Dos Santos Filho 2007
Orchidaceae	*Acianthera sonderana*	70.00	0.20	3.89	0.25	2.96, 4.82	-49.258889	-25.490833	Dos Santos Filho 2007
Orchidaceae	*Cirrhaea dependens*	68.18	0.90	3.02	0.02	2.35, 3.68	-46.866667	-23.183333	Pansarin et al. 2006
Orchidaceae	*Corymborkis flava*	78.57	66.67	0.33	0.10	-0.22, 0.89	-42.858611	-20.801944	Vieira et al. 2007
Orchidaceae	*Elleanthus brasiliensis*	75.76	30.39	1.09	0.20	0.76, 1.41	-44.837500	-23.356111	Nunes 2011
Orchidaceae	*Elleanthus crinipes*	64.71	16.73	1.22	0.08	0.64, 1.80	-45.223056	-23.435556	Nunes 2011
Orchidaceae	*Epidendrum secundum*	80.00	7.54	2.15	0.10	1.69, 2.60	-46.866667	-23.183333	Pansarin and Amaral 2008a
Orchidaceae	*Gongora quinquenervis*	72.73	1.05	3.05	0.47	2.35, 3.74	-34.953333	-7.812500	Martini et al. 2003
Orchidaceae	*Govenia utriculata*	63.33	10.07	1.51	0.13	1.00, 2.01	-46.866667	-23.250000	Pansarin 2008b
Orchidaceae	*Grobya amherstiae*	82.00	33.78	1.21	0.24	0.80, 1.62	-46.866667	-23.250000	Mickeliunas et al. 2006
Orchidaceae	*Polystachya estrellensis*	68.75	87.46	-0.64	0.14	-1.23, -0.04	-46.866667	-23.250000	Pansarin and Amaral 2006
Orchidaceae	*Psilochilus modestus*	90.24	19.47	2.01	0.21	1.39, 2.63	-46.866667	-23.183333	Pansarin and Amaral 2008b
Orchidaceae	*Stanhopea insignis*	90.48	25.37	1.84	0.11	0.98, 2.69	-45.066667	-23.550000	Pansarin and Amaral 2009
Orchidaceae	*Stanhopea lietzei*	65.00	16.85	1.22	0.06	0.63, 1.81	-46.866667	-23.183333	Pansarin and Amaral 2009
Orobanchaceae	*Velloziela dracocephaloides*	75.00	46.15	0.69	0.42	-0.60, 1.99	-44.600000	-22.450000	Wolowski 2013

Table S1. Continued.

Family	Species	CP	NP	*d*	*v*	95% CI	Longitude	Latitude	Reference
Passifloraceae	*Mitostemma glaziovii*	95.65	23.53	2.35	0.12	1.09, 3.62	-41.066667	-21.400000	Benevides 2006
Passifloraceae	*Passiflora alata*	50.00	27.78	0.53	0.20	-0.36, 1.42	-41.066667	-21.400000	Benevides 2006
Passifloraceae	*Passiflora miersii*	60.00	51.85	0.18	0.03	-0.89, 1.25	-47.100000	-22.816667	Koschnitze and Sazima 1997
Passifloraceae	*Passiflora suberosa*	91.67	52.94	1.26	0.06	0.01, 2.50	-47.100000	-22.816667	Koschnitze and Sazima 1997
Podostemaceae	*Mourera fluviatillis*	88.33	92.50	-0.27	0.10	-0.90, 0.36	-35.583333	-8.550000	Sobral-Leite et al. 2011
Rubiaceae	*Bathysa australis*	17.50	28.00	-0.33	0.22	-0.81, 0.15	-42.283333	-22.525	Andrich 2008
Rubiaceae	*Bathysa cuspidata*	22.50	22.00	0.02	0.04	-0.43, 0.46	-44.600000	-22.450000	Andrich 2008
Rubiaceae	*Chiococca alba*	61.54	53.49	0.18	0.08	-0.30, 0.67	-44.833333	-23.350000	Castro et al. 2008
Rubiaceae	*Manettia luteo-rubra*	89.29	90.00	-0.04	0.78	-1.08, 1.00	-48.533333	-24.183333	Passos and Sazima 1995
Rubiaceae	*Manettia mitis*	70.31	50.00	0.48	0.04	0.12, 0.83	-44.600000	-22.450000	Wolowski 2013
Rubiaceae	*Palicourea longepedunculata*	71.43	24.40	1.13	0.04	0.61, 1.65	-42.858611	-20.801944	Silva et al. 2010
Rubiaceae	*Psychotria barbiflora*	59.38	20.38	0.96	0.09	0.54, 1.38	-34.875000	-8.125000	Teixeira and Machado 2004a
Rubiaceae	*Psychotria birotula*	35.21	40.13	-0.12	0.02	-0.44, 0.21	-44.833333	-23.350000	Castro et al. 2004
Rubiaceae	*Psychotria brasiliensis*	56.52	41.77	0.33	0.14	-0.08, 0.73	-44.215000	-23.182500	Almeida 2005
Rubiaceae	*Psychotria mapourioides*	27.12	26.67	0.01	0.07	-0.43, 0.46	-44.833333	-23.350000	Castro et al. 2004
Rubiaceae	*Psychotria nuda*	23.53	20.83	0.12	0.73	-0.22, 0.46	-44.215000	-23.182500	Almeida 2005
Rubiaceae	*Psychotria suterella*	62.07	54.55	0.17	0.18	-0.34, 0.68	-47.000000	-23.708333	Lopes and Buzato 2005
Rubiaceae	*Psychotria tenuinervis*	12.90	19.57	-0.47	0.06	-1.24, 0.30	-42.466667	-22.833333	Virillo et al. 2007
Rubiaceae	*Rudgea jasminoides*	71.08	49.41	0.51	0.03	0.16, 0.86	-44.833333	-23.350000	Castro et al. 2004

Table S1. Continued.

Family	Species	CP	NP	*d*	*v*	95% CI	Longitude	Latitude	Reference
Rubiaceae	*Sabicea cinerea*	52.38	60.00	-0.17	0.44	-0.85, 0.51	-34.875000	-8.125000	Teixeira and Machado 2004b
Rutaceae	*Galipea jasminiflora*	47.52	3.14	1.84	0.03	1.43, 2.24	-47.100000	-22.816667	Piedade and Ranga 1993
Rutaceae	*Metrodorea nigra*	25.93	5.88	0.95	0.13	0.43, 1.47	-47.100000	-22.816667	Pombal and Morellato 2000
Rutaceae	*Metrodorea stipularis*	35.71	16.00	0.59	0.08	-0.08, 1.26	-47.100000	-22.816667	Pombal and Morellato 2000
Solanaceae	*Dyssochroma viridiflora*	95.00	92.03	0.27	0.30	-0.58, 1.13	-43.316667	-22.916667	Verçoza 2003
Verbenaceae	*Citharexylum myrianthum*	100.00	0.00	6.21	0.15	4.04, 8.38	-44.833333	-23.350000	Rocca and Sazima 2006
Violaceae	*Amphirrhox longifolia*	20.00	10.96	0.39	0.03	-0.88, 1.66	-34.990278	-7.798056	Braun 2010
Violaceae	*Hybanthus atropurpureus*	54.55	52.14	0.05	0.06	-0.32, 0.43	-47.109167	-22.829722	Rosero-Lasprilla 1997
Violaceae	*Paypayrola blanchetiana*	8.14	1.04	1.17	0.07	0.69, 1.66	-34.990278	-7.798056	Braun 2010

References

Agostini K (2008) Ecologia da reprodução de duas espécies de Mucuna (Leguminosae, Faboideae, Phaseoleae) - embriologia, citogenética e variabilidade genética - do litoral norte de São Paulo. PhD Thesis, State University of Campinas, Campinas.

Almeida EM (2005) Ecologia reprodutiva e comportamento dos visitantes florais e dos frugívoros de duas espécies de *Psychotria* L. (Rubiaceae) em uma área de Floresta Atlântica, Ilha grande, Rio de Janeiro. PhD thesis, Federal University of Rio de Janeiro, Rio de Janeiro.

Andrich M (2008) Sistema reprodutivo e polinização em duas espécies arbóreas e simpátricas de *Bathysa* (Rubiaceae). Master's dissertation, Botanical Garden of Rio de Janeiro, Rio de Janeiro.

Araujo AC, Fischer EA, Sazima M (1994) Floração sequencial e polinização de três espécies de *Vriesea* (Bromeliaceae) na região de Juréia, sudeste Brasil. Rev Bras Bot 17(2): 113-118.

Barreto AA, Freitas L (2007) Atributos florais em um sistema de polinização especializado: *Calathea cylindrica* (Roscoe) K. Schum. (Marantaceae) e abelhas Euglossini. Rev Bras Bot 30(3): 421-431.

Benevides CR (2006) Biologia floral e polinização de Passifloraceae nativas e cultivadas na região Norte Fluminense-RJ. Master's dissertation, State University of North Fluminense, Campos dos Goytacazes.

Borges HBN (1991) Biologia reprodutiva de quatro espécies de Melastomataceae. Master's dissertation thesis, State University of Campinas, Campinas.

Borges LA, Sobrinho MS, Lopes AV (2009) Phenology, pollination, and breeding system of the threatened tree *Caesalpinia echinata* Lam. (Fabaceae), and a review of studies on here productive biology in the genus. Flora 204(2): 111-130.

Braun M (2010) Floral biology, breeding system, pollination and reproductive success of selected understory tree species in fragments of Atlantic Rainforest in Pernambuco, Northeast Brazil. PhD thesis, University of Ulm, Ulm.

Castro CC, Oliveira PEAM, Alves MC (2004) Breeding system and floral morphometry of distylous *Psychotria* L. species in the Atlantic Rain Forest, SE Brazil. Plant Biology 6(6): 755-760.

Castro CC, Oliveira PEAM, Pimentel RMM (2008) Reproductive biology of the herkogamous vine *Chiococca alba* (L.) Hitchc. (Rubiaceae) in the Atlantic Rain Forest, SE Brazil. Rev Bras Bot 31(2): 317-321.

Dos Santos Filho JF (2007) Polinização e biologia reprodutiva de três espécies do gênero *Acianthera* Scheidw. (Orchidaceae) em floresta ombrófila mista. Master's dissertation, Federal University of Parana, Curitiba.

Franco ALM (1991) Biologia floral de duas espécies sincronopátricas de *Prestonia* (Apocynaceae). Master's dissertation thesis, State University of Campinas, Campinas.

Franco ALM (1995) Ecologia da polinização e biologia reprodutiva de sete espécies de Phaseoleae (Fabaceae). PhD thesis, State University of Campinas, Campinas.

Franco AM (2007) Biologia reprodutiva de três espécies de Melastomataceae (*Tibouchina cerastifolia* Cong., *T. clinopodifolia* Cong. E *T. gracilis* Cong.) nos mananciais da serra, Piraquara, Paraná. Master's dissertation, Federal University of Parana, Curitiba.

Goldenberg R, Varassin IG (2001) Sistemas reprodutivos de espécies de Melastomataceae da Serra do Japi. Jundiaí. São Paulo. Brasil. Rev Bras Bot 24(3): 283-288.

Koschnitze C, Sazima M (1997) Biologia floral de cinco espécies de *Passiflora* L. (Passifloraceae) em mata semidecídua. Rev Bras Bot 20(2): 119-126.

Lima NAS, Vieira MF (2006) Fenologia de floração e sistema reprodutivo de três espécies de *Ruellia* (Acanthaceae) em fragmento florestal de Viçosa, sudeste

brasileiro. Rev Bras Bot 29(4): 681-687.

Lopes AV, Machado IC (1998). Floral biology and reproductive ecology of *Clusia nemorosa* (Clusiaceae) in northeastern Brazil. Plant Syst Evol 213: 71-90.

Lopes AV, Machado IC (1999) Pollination and reproductive biology of *Rauvolfia grandiflora* (Apocynaceae): secondary pollen presentation. herkogamy and self-incompatibility. Plant Biology 1(5): 547-553.

Lopes AVF, Machado ICS (1996) Biologia floral de *Swartzia pickelii* Killip ex Ducke (Leguminosae - Papilionoideae) e sua polinização por *Eulaema* spp. (Apidae - Euglossini). Rev Bras Bot 19(1): 17-24.

Lopes LE, Buzato S (2005) Biologia reprodutiva de *Psychotria suterella* Muell. Arg. (Rubiaceae) e a abordagem de escalas ecológicas para a fenologia de floração e frutificação. Rev Bras Bot 28(4): 785-795.

Machado IC, Loiola MI (2000) Fly pollination and pollinator sharing in two synchronopatric species: *Cordia multispicata* (Boraginaceae) and *Borreria alata* (Rubiaceae). Rev Bras Bot 23(3): 305-311.

Machado ICS, Sazima I, Sazima M (1998) Bat pollination of the terrestrial herb *Irlbachia alata* (Gentianaceae) in northeastern Brazil. Plant Syst Evol 209(3-4): 231-237.

Martinelli G (1997) Biologia reprodutiva de Bromeliaceae na Reserva Ecológica de Macaé de Cima. In: de Lima HC, Guedes-Bruni RR, editors. Serra da Macaé de Cima: Diversidade Florística e Conservação da Mata Atlântica. Rio de Janeiro: Jardim Botânico do Rio de Janeiro. pp. 213-250.

Martini P, Schlindwein C, Montenegro A (2003) Pollination flower longevity and reproductive biology of *Gongora quinquenervis* Ruiz and Pavon (Orchidaceae) in an Atlantic Forest fragment of Pernambuco, Brazil. Plant Biology 5(5): 496-503.

Mello RB (2004) Biologia da reprodução de duas espécies de Campanulaceae Jussieu em Nova Friburgo, RJ. Master's dissertations, Federal University of Rio de Janeiro, Rio de Janeiro.

Melo GFA, Machado IC (1998) Autoincompatibilidade em *Miconia ciliata* (L.C.Rich) Dc. (Miconieae-Melastomataceae). Acta Bot Bras 12: 113-120.

Mickeliunas L, Pansarin ER, Sazima M (2006) Biologia floral, melitofilia e influência de besouros Curculionidae no sucesso reprodutivo de *Grobya amherstiae* Lindl. (Orchidaceae: Cyrtopodinae). Rev Bras Bot 29(2): 251-258.

Nunes CEP (2011) Biologia da polinização e reprodução de *Elleanthus* C.Presl. (Orchidaceae) na Mata Atlântica do Parque Estadual da Serra do Mar, Sao Paulo. Master's dissertation, State University of Campinas, Campinas.

Cruz Neto O (2007) Fenologia, biologia reprodutiva, eficiência dos visitantes florais de espécies simpátricas de *Inga* (Leguminosae - Mimosoideae) em remanescente de Floresta Atlântica no nordeste do Brasil. Master's dissertation, Federal University of Pernambuco, Recife.

Pansarin ER, Amaral MCE (2006) Biologia reprodutiva e polinização de duas espécies de *Polystachya* Hook. no Sudeste do Brasil: evidência de pseudocleistogamia em Polystachyeae (Orchidaceae). Rev Bras Bot 29(3): 423-432.

Pansarin ER, Bittrich V, Amaral MCE (2006) At daybreak - Reproductive biology and isolating mechanisms of *Cirrhaea dependens* (Orchidaceae). Plant Biology 8(4): 494-502.

Pansarin ER (2008a) Reproductive biology of *Echinodorus longipetalus* (Alismataceae): sexual morphs, breeding system and pollinators. Aquat Bot 89(4): 404-408.

Pansarin ER (2008b) Reproductive biology and pollination of *Govenia utriculata*: A syrphid fly orchid pollinated through a pollen-deceptive mechanism. Plant Spec Biol 23(2): 90-96.

Pansarin ER, Amaral MCE (2008a) Reproductive biology and pollination mechanisms of *Epidendrum secundum* (Orchidaceae). Floral variation: a consequence of natural hybridization? Plant Biology 10(2): 211-219.

Pansarin ER, Amaral MCE (2008b) Pollen and nectar as a reward in the basal epidendroid *Psilochilus modestus* (Orchidaceae: Triphoreae): A study of floral morphology. reproductive biology and pollination strategy. Flora 203: 474-483.

Pansarin ER, Amaral MCE (2009) Reproductive biology and pollination of southeastern Brazilian *Stanhopea* Frost ex Hook. (Orchidaceae). Flora 204(3): 238-249.

Passos LC (1995) Fenologia, polinização e reprodução de duas espécies de *Croton* (Euphorbiaceae) em Mata Semidecídua. Master's dissertation, State University of Campinas, Campinas.

Passos L, Sazima M (1995) Reproductive biology of the distylous *Manettia luteo-rubra* (Rubiaceae). Bot Acta 108(4): 309-313.

Pereira MFPA (1998) Biologia da polinização de duas espécies de Acanthaceae, na região de Campinas, São Paulo. Master's dissertation, State University of Campinas, Campinas.

Piedade LH, Ranga NT (1993) Ecologia da Polinização de *Galipea jasminiflora* Engler (Rutaceae). Rev Bras Bot 16(2): 151-157.

Pires JPA, Freitas L (2008) Reproductive biology of two tree species of Leguminosae in a Montane Rain Forest in southeastern Brazil. Flora 203(6): 491-498.

Pombal ECP, Morellato LPC (2000) Differentiation of floral color and odor in two fly pollinated species of *Metrodorea* (Rutaceae) from Brazil. Plant Syst Evol 221(3-4): 141-156.

Rocca M, Sazima M (2006) The dioecious. sphingophilous species *Citharexylum myrianthum* (Verbenaceae): Pollination and visitor diversity. Flora 201(6): 440-450.

Rosero-Lasprilla L (1997) Biologia reprodutiva de *Hybanthus atropurpureus* (St. Hil.) Taub. da Reserva Municipal de Santa Genebra, Sudeste Brasil. Master's dissertation, State University of Campinas, Campinas.

San-Martin Gajardo I, Freitas L (1999) Hummingbird pollination in *Besleria longimucronata* Hoehne (Gesneriaceae) in Southeastern Brazil. Biociências 7: 13-24.

San-Martina Gajardo I (2004) Biologia da polinização de espécies da tribo Sinningieae (Gesneriaceae) no sudeste do Brasil. PhD thesis, State University of Campinas, Campinas.

Sazima M, Sazima I (1985) Biologia floral de *Dalechampia stipulacea* (Euphorbiaceae) e sua polinização por *Euglossa melanotricha* (Apidae). Rev Bras Bot 45(1/2): 85-93.

Sigrist MR, Sazima M (2002) *Ruellia brevifolia* (Pohl) Ezcurra (Acanthaceae): fenologia da floração. biologia da polinização e reprodução. Rev Bras Bot 25(1): 35-42.

Sigrist MR, Sazima M (2004) Pollination and reproductive biology of twelve species of neotropical malpighiaceae: Stigma morphology and its implications for the breeding system. Ann Bot-London 94(1): 33-41.

Sigrist MR (1992) Fenologia, biologia flora, sistema de polinização e sistema reprodutivo de duas espécies simpátricas de *Dichorisandra* (Commelinaceae). Master's dissertation, State University of Campinas, Campinas.

Silva CA, Vieira MF, Amaral CH (2010) Floral attributes, ornithophily and reproductive success of *Palicourea longepedunculata* (Rubiaceae), a distylous shrub in southeastern Brazil. Rev Bras Bot 33(2): 207-210.

Siqueira Filho JA (2003) Fenologia da floração, ecologia da polinização e conservação de Bromeliaceae na Floresta Atlântica Nordestina. PhD thesis, Federal University of Pernambuco, Recife.

Sobral-Leite M, Siqueira Filho JA, Erbar C, Machado IC (2011) Anthecology and reproductive system of *Mourera fluviatilis* (Podostemaceae): Pollination by bees and xenogamy in a predominantly anemophilous and autogamous family? Aquat Bot 95: 77-87.

Teixeira LAG, Machado IC (2000) Sistema de Polinização e reprodução de *Byrsonima sericea* DC (Malpighiaceae). Acta Bot Bras 14(3): 347-357.

Teixeira LAG, Machado IC (2004a) Biologia da polinização e sistema reprodutivo de *Psychotria barbiflora* DC. (Rubiaceae). Acta Bot Bras 18(4): 853-862.

Teixeira LAG, Machado IC (2004b) *Sabicea cinerea* Aubl. (Rubiaceae): distilia e polinização em um fragmento de floresta Atlântica em Pernambuco. Nordeste do Brasil. Rev Bras Bot 27(1): 193-204.

Teixeira LAG (2005) Mecanismos de polinização e sistema reprodutivo de espécies de Marantaceae da Estação Ecológica do Tapacurá, Pernambuco,

Nordeste do Brasil. PhD thesis, Federal University of Pernambuco, Recife.

Verçoza FC (2003) Fenologia e biologia reprodutiva de *Dyssochroma viridiflora* Miers var. *viridiflora* (Solanaceae). Master's dissertation, Federal University of Rio de Janeiro, Rio de Janeiro.

Vieira MF, Grabalos R (2003) Sistema reprodutivo de *Oxypetalum mexiae* Malme (Asclepiadaceae), espécie endêmica de Viçosa. MG. Brasil, em perigo de extinção. Acta Bot Bras 17(1): 137-145.

Vieira MF, Andrade MRS, Bittencourt NS, de Carvalho-Okano RM (2007) Flowering phenology, nectary structure and breeding system in *Corymborkis flava* (Spiranthoideae: Tropidieae), a terrestrial orchid from a Neotropical forest. Aust J Bot 55(6): 635-642.

Virillo CB, Ramos FN, Castro CC, Semir J (2007) Floral biology and breeding system of *Psychotria tenuinervis* Muell. Arg. (Rubiaceae) in the Atlantic rain forest. SE Brazil. Acta Bot Bras 21(4): 879-884.

Wolowski M, Freitas L (2010) Sistema reprodutivo e polinização de *Senna multijuga* (Fabaceae) em Mata Atlântica Montana. Rodriguésia 61(2): 167-179.

Wolowski M (2013) Sistema reprodutivo e limitação polínica em plantas da Floresta Atlântica com ênfase em polinização por beija-flores. PhD thesis, Rio de Janeiro.
