# Supplementary material for: Meta-Analysis of Pollen Limitation Reveals the Relevance of Pollination Generalization in the Atlantic Forest of Brazil
Source: PLoS One. 2014 Feb 21;9(2):e89498. doi: 10.1371/journal.pone.0089498 (PMC3931788; doi:10.1371/journal.pone.0089498)
Supplement: Table S2 — Results of traditional and phylogenetically independent meta-analyses based on random-effects models. Heterogeneity between categories (Qb), degrees of freedom (df), P value, and Akaike's information criterion (AIC) per plant feature. Effect size (d) and 95% confidence interval (95% CI), Z value, degrees of freedom (df), and P value per category of plant feature. (PDF) [file pone.0089498.s003.pdf]

Table S2. Results of traditional and phylogenetically independent meta-analyses based on random-effects models. Heterogeneity between categories (Q_b), degrees of freedom (*df*), *P* value, and Akaike's information criterion (AIC) per plant feature. Effect size (*d*) and 95% confidence interval (95% CI), Z value, degrees of freedom (*df*), and *P* value per category of plant feature.

Plant feature	Number of species	Traditional meta-analysis									Phylogenetically-independent meta-analysis								
		Q_b	*df*	*P*	AIC	*d*	95% CI	Z	*df*	*P*	Q_b	*df*	*P*	AIC	*d*	95% CI	Z	*df*	*P*
**Pollinator species**	**103**	**5.74**	**1**	**0.17**	**301.50**						**4.80**	**1**	**0.03**	**305.19**					
1	24					0.79	0.51,1.06	31.08	1	<0.001					0.84	0.55,1.13	32.04	1	<0.001
2 - 5	40					0.36	0.14,0.57	10.50	1	0.0012					0.38	0.14,0.62	9.38	1	0.0022
> 5	39					0.50	0.28,0.72	20.40	1	<0.001					0.50	0.27,0.74	17.24	1	<0.001
**Flower symmetry**	**121**	**3.72**	**1**	**0.05**	**358.03**						**3.56**	**1**	**0.06**	**360.30**					
Zygomorphic	68					0.61	0.45,0.77	54.02	1	<0.001					0.73	0.54,0.94	59.39	1	<0.001
Actinomorphic	53					0.37	0.19,0.55	15.78	1	<0.001					0.39	0.18,0.59	13.1	1	<0.001
**Reward**	**121**	**0.73**	**1**	**0.39**	**360.20**						**0.30**	**1**	**0.58**	**362.80**					
Nectariferous [nectar]	70					0.47	0.31,0.63	33.02	1	<0.001					0.47	0.28,0.65	24.56	1	<0.001
Nectariferous [nectar and others]	16					0.48	0.15,0.80	8.31	1	0.0039					0.44	0.09,0.78	6.27	1	0.0123
Nectarless [others]	35					0.59	0.37,0.83	25.63	1	<0.001					0.59	0.34,0.84	22.03	1	<0.001
**Pollinator functional group**	**121**	**2.33**	**1**	**0.13**	**362.81**						**1.85**	**1**	**0.17**	**365.38**					
1	90					0.51	0.37,0.65	51.68	1	<0.001					0.53	0.37,0.69	41.23	1	<0.001
2	23					0.62	0.35,0.80	19.81	1	<0.001					0.68	0.38,0.97	20.55	1	<0.001
≥ 3	8					0.23	-0.21,0.66	1.01	1	0.3747					0.23	-0.22,0.68	1.03	1	0.3111
**Pollinator group**	**121**	**0.86**	**1**	**0.34**	**363.52**						**1.27**	**1**	**0.26**	**365.31**					
Vertebrate	45					0.49	0.30,0.69	24.53	1	<0.001					0.51	0.29,0.74	19.83	1	<0.001
Invertebrate	67					0.49	0.33,0.65	36.43	1	<0.001					0.49	0.31,0.67	27.99	1	<0.001
Mixed	9					0.71	0.26,1.16	9.73	1	0.0018					0.74	0.27,1.21	9.58	1	0.0020
**Mating system**	**123**	**1.9**	**1**	**0.17**	**367.08**						**2.13**	**1**	**0.14**	**367.72**					
Self-compatible	69					0.44	0.28,0.59	29.56	1	<0.001					0.50	0.31,0.68	27.91	1	<0.001
Self-incompatible	54					0.60	0.43,0.77	46.64	1	<0.001					0.62	0.43,0.81	39.76	1	<0.001
**Autogamy**	**95**	**1.74**	**1**	**0.19**	**285.82**						**1.42**	**1**	**0.23**	**287.47**					
Autogamous	15					0.29	-0.09,0.66	2.24	1	0.1344					0.45	0.05,0.85	4.93	1	0.0265
Non-autogamous	80					0.56	0.41,0.70	56.55	1	<0.001					0.56	0.39,0.73	42.82	1	<0.001
**Plant habit**	**126**	**2.96**	**1**	**0.09**	**380.57**						**1.78**	**1**	**0.18**	**383.70**					
Herbs	40					0.58	0.37,0.78	30.97	1	<0.001					0.54	0.30,0.77	19.29	1	<0.001
Woody plants	63					0.40	0.25,0.56	25.86	1	<0.001					0.46	0.28,0.65	24.42	1	<0.001
Vines	23					0.64	0.36,0.78	19.94	1	<0.001					0.72	0.41,1.03	21.25	1	<0.001
